# CD4 response of QuantiFERON-TB Gold Plus for positive consistency of latent tuberculosis infection in patients on dialysis

**DOI:** 10.1038/s41598-020-78374-3

**Published:** 2020-12-07

**Authors:** Ping-Huai Wang, Shu-Yung Lin, Susan Shih-Jung Lee, Shu-Wen Lin, Chih-Yuan Lee, Yu-Feng Wei, Chin-Chung Shu, Jann-Yuan Wang, Chong-Jen Yu

**Affiliations:** 1 Division of Pulmonology, Department of Internal Medicine, Far Eastern Memorial Hospital, New Taipei City, Taiwan; 2 Department of Nursing, Oriental Institute of Technology, New Taipei City, Taiwan; 3 Department of Internal Medicine, National Taiwan University Hospital, No. 7, Chung Shan South Road, Taipei, Taiwan; 4 College of Medicine, National Taiwan University, Taipei, Taiwan; 5 Division of Infectious Diseases, Department of Internal Medicine, Kaohsiung Veterans General Hospital, Kaohsiung, Taiwan; 6 Faculty of Medicine, School of Medicine, National Yang-Ming University, Taipei, Taiwan; 7 Graduate Institute of Clinical Pharmacy, National Taiwan University, Taipei, Taiwan; 8 Department of Surgery, National Taiwan University Hospital, Taipei, Taiwan; 9 Division of Chest Medicine, Department of Internal Medicine, E-Da Hospital, Kaohsiung, Taiwan

**Keywords:** Biomarkers, Health care, Medical research, Risk factors

## Abstract

A significantly negative reversion in the QuantiFERON-TB Gold In-tube (QFT-GIT) test is reported in patients on dialysis, which makes the results unreliable. The CD4 and CD8 responses of the QFT-Gold plus (QFT-Plus) may have better positive consistency, but this needs to be investigated. We enrolled dialysis patients with baseline positive QFT-GIT_0_ results and conducted two rounds of follow-up paired QFT-GIT_1&2_ and QFT-Plus_1&2_ tests at an interval of 6 months. The positive consistency, concordance, and discordance of the QFT results were analyzed. A total of 236 patients on dialysis were screened, and 73 participants with positive QFT-GIT_0_ results were enrolled. The baseline QFT-GIT_0_ response was higher in the 1st QFT-Plus_1_(+) group than in the QFT-Plus_1_(−) group, but insignificantly different between the 1st QFT-GIT_1_(+) and QFT-GIT_1_(−) groups. The two assays had good correlation when concurrently tested. Fifty-three subjects completed a second round of the QFT-GIT_2_ and QFT-Plus_2_. Persistent positivity was higher with the QFT-Plus_2_ (81.8%) than with the QFT-GIT_2_ (58.8%, p = 0.040). The QFT-GIT_1_ and QFT-Plus_1_ CD4 responses were higher in patients with persistent positivity than in those with negative reversion, whereas the difference of the QFT-Plus TB1 and TB2 data, representative of the CD8 response, were similar between positive persistence and negative reversion. In conclusion, the QFT-Plus provides more reliable positive consistency than does the QFT-GIT. The CD4 interferon-γ response might play a role in maintaining positivity of LTBI.

## Introduction

Tuberculosis (TB) remains one of the most common infectious diseases in the world. According to the World Health Organization, around 10.0 million TB cases and an estimated 1.2 million TB deaths occurred in HIV-negative people in 2018^1^. In fact, an estimated one quarter of the world’s people carry *Mycobacterium tuberculosis* (M.tb)^2^. When elderly and immunocompromised patients develop active TB, mortality and morbidity are still high^3,4^. In the future of the management of active TB, latent TB infection (LTBI) intervention is one strategy for preventing reactivation and reducing transmission^5,6^.

Among the high-risk populations for TB reactivation, patients on dialysis have a higher incidence of TB (10–25 times higher) than the general population^7,8^. Notably, dialysis patients have high mortality once active TB develops due to the atypical manifestation and delayed diagnosis^9,10^. Therefore, in addition to those with close contact with TB, patients receiving dialysis are also highly prioritized for LTBI intervention in Taiwan^5^.

However, LTBI is diagnosed indirectly by immune assays such as the tuberculin skin test and interferon-gamma release assays (IGRAs), which, due to immune depression, sometimes yield false negative results in patients receiving dialysis^11,12^. Although our prior study using QuantiFERON-TB Gold In-tube (QFT-GIT) demonstrated an acceptable indeterminate rate of < 5% in dialysis patients^12^, the negative reversion rate at 6-month follow-up was 44–48%^13,14^, which was higher than the 33–35% in contacts or healthcare workers^15,16^. Notably, the greater consistency of positive QFT-GIT tests, a kind of IGRA, is reportedly correlated with the higher incidence of TB in patients receiving dialysis^14^. The hazard ratio for TB development increases by 10.4- to 14.4-fold if QFT-GIT results remain positive after 6 months^14^.

A test with better consistency of positive results is needed because serial follow-up using IGRA is time-consuming and wasteful of resources. Currently, a new test, the QuantiFERON-TB Gold Plus (QFT-Plus), has modified epitopes for CD4 and CD8 lymphocyte-specific reactions. Better LTBI diagnosis and lower non-specific reactions have been reported^17–20^. But all the reports to date have been based on cross-sectional designs, either for active TB or for LTBI, and they have lacked discussion of immunocompromised patients. Although the QFT-Plus might have a more specific M.tb response and have positive consistency, it still needs to be validated. In the present study, we aimed to examine the consistency of positive LTBI status after 6 months by directly comparing QFT-Plus and QFT-GIT assays in dialysis patients.

## Results

### Participant enrollment

**Figure 1:**
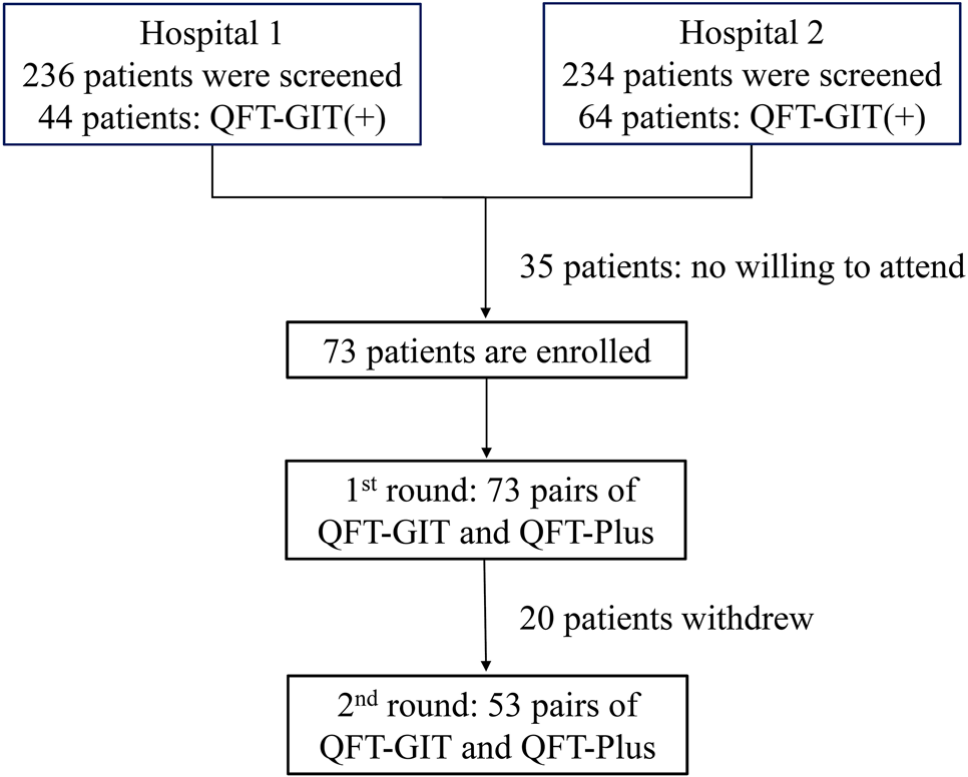
Flowchart of participant enrollment. QFT-GIT, QuantiFERON-TB Gold In-tube; QFT-Plus, QuantiFERON-TB Gold Plus.

A total of 236 patients on maintenance dialysis at hospital 1 and 234 patients of hospital 2 were screened for LTBI (Fig. 1). Among them, 108 patients (44 [18.6%] from hospital 1 and 64 [27.4%] from hospital 2) had positive QFT-GIT results. Thirty-five of these patients rejected the invitation to participate in the study. Thereafter, 73 subjects were enrolled in the first round of testing of the QFT-GIT and QFT-Plus together. Around 6 months later, at the follow-up testing of the QFT-GIT and QFT-Plus, twenty participants withdrew from the study, leaving fifty-three participants in the second round.

### Baseline test of QFT-GIT_0_ and first follow-ups of QFT-GIT_1_ and QFT-Plus_1_

The 73 participants were 56.7 ± 11.2 years old (mean ± standard deviation). The majority were men (71%) and patients receiving hemodialysis (80.8%) (Table 1). After the 1st round of paired tests, fifty-seven participants received LTBI treatment during follow-up, with similar distributions of QFT-GIT_1_(+) and QFT-Plus_1_(+) results. Weekly high-dose rifapentine plus isoniazid for 3 months was prescribed to 74%, and 9 months of isoniazid daily were prescribed to the remaining 26%. The mean IFN-γ response of the baseline QFT-GIT (QFT-GIT_0_) was 2.31 ± 2.40 IU/ml for all. The first round of follow-up QFT-GIT and QFT-Plus results were QFT-GIT(+) for 49 subjects (67.1% of 73 patients) and QFT-Plus(+) for 47 subjects (64.4%). The demographic data, including BCG scar and QFT-GIT_0_, were not significantly different between the first QFT-GIT(+) and QFT-Plus (+) groups. The QFT-GIT_0_ response was higher in the QFT-Plus_1_(+) group than in the QFT-Plus_1_(−) group (2.93 vs. 1.18 IU/ml, p = 0.002). By contrast, the QFT-GIT_0_ response was not significantly different between the QFT-GIT_1_(+) and QFT-GIT_1_(−) cases (2.65 vs. 1.60 IU/ml, p = 0.080) (Table S1, supplement file).

**Table 1:** Demographic data of study population.

	Total (n = 73)	1st QFT-GIT(+) (n = 49)	1st QFT-Plus(+) (n = 47)	p value^‡^
Age	56.7 ± 11.2	57.7 ± 11.1	57.4 ± 11.6	0.766
Sex, male n (%)	52 (71)	35 (71)	33 (70)	1
Dialysis, HD* n (%)	59 (81)	36 (73)	37 (79)	0.635
DM n (%)	15 (21)	12 (25)	14 (30)	0.648
Rheumatoid n (%)	1 (1)	1 (2)	1 (2)	1
Previous TB n (%)	4 (6)	2 (4)	3 (6)	0.674
BCG scar^#^ n (%)	65 (89)	44 (90)	41 (97)	0.757
QFT-GIT_0_^†^_,_ IU/ml	2.31 ± 2.40	2.65 ± 2.36	2.93 ± 2.38	0.914
LTBI treatment n (%)	57 (75)	38 (78)	35 (75)	0.182

Data are presented as number (percentage) or mean ± standard deviation.

None had underlying cancer or cirrhosis.

BCG, Bacillus Calmette-Guérin vaccine; DM, diabetes mellitus; M, male; HD, hemodialysis; LTBI, latent tuberculosis infection; QFT-GIT, QuantiFERON-TB Gold In-Tube; QFT-Plus, QuantiFERON-TB Gold Plus; TB, tuberculosis.

*Regarding dialysis modality, those not receiving hemodialysis received peritoneal dialysis.

^#^Missing data of BCG: four patients in the total population; two patients in the 1st QFT-GIT(+) group and two in the 1st QFT-Plus(+) group, respectively.

^†^QFT-GIT_0_: the interferon-gamma level of the TB Antigen tube minus the negative control tube at the baseline test of the QuantiFERON-TB Gold In-Tube.

^‡^p values were compared between patients with 1st QFT-GIT(+) and those with 1st QFT-Plus(+).

As shown in Table 2, for the IFN-γ response of the first follow-up test of the QFT-GIT (QFT-GIT_1_), the TB1 tube in the first test of the QFT-Plus (T1 of QFT-Plus_1_) and the TB2 tube in the first test of the QFT-Plus_1_ (T2 of QFT-Plus_1_) had good correlation with the IFN-γ response of the QFT-GIT_0_ (Spearman correlation r: 0.520, 0.611, and 0.611, respectively [all p < 0.001]). However, the QFT-GIT_0_ had no significant correlation with the IFN-γ level of the TB2 tube minus the TB1 tube of the QFT-Plus (T2–T1 of the QFT-Plus_1_) (Spearman correlation coefficient r: 0.085, p = 0.477).

**Table 2:** The associations (1) between the interferon-gamma level of QuantiFERON-TB Gold In-tube (QFT-GIT) at baseline and at 1st follow-up with the QFT-GIT and QuantiFERON-TB Gold Plus (QFT-Plus) and (2) between the two tests at 1st follow-up.

	mean ± SD	p	Spearman coefficient
**(1) QFT-GIT**_0_*	2.31 ± 2.40		
versus QFT-GIT_1_*	1.49 ± 1.97	< 0.001	0.520
versus T1 of QFT-Plus_1_*	1.30 ± 1.80	< 0.001	0.611
versus T2 of QFT-Plus_1_*	1.36 ± 1.92	< 0.001	0.611
versus T2–T1 of QFT-Plus_1_*	0.064 ± 0.513	0.477	0.085
**(2) QFT-GIT**_1_*	1.49 ± 1.97		
versus T1 of QFT-Plus_1_*	1.30 ± 1.80	< 0.001	0.879
versus T2 of QFT-Plus_1_*	1.36 ± 1.92	< 0.001	0.895
versus T2–T1_1_ of QFT-Plus_1_*	0.064 ± 0.513	0.206	0.150

QFT-GIT response: the interferon-gamma level of the TB antigen tube minus the negative control.

T1 or T2 response: the interferon-gamma level of the TB1 or TB2 tube, respectively, minus the negative control of the QFT-Plus test.

All QFT responses are represented as IU/ml.

*0: baseline test; 1: 1st follow-up, around 6 months later.

### Discordance and concordance of first follow-up QFT-Plus and QFT-GIT tests

Regarding the QFT_1_ response, the QFT-GIT_1_ had good correlation with T1 of the QFT-Plus_1_ and T2 of the QFT-Plus_1_ (r = 0.879 and 0.895, respectively, both p < 0.001) (Table 2). But T2–T1 of the QFT-Plus_1_ had non-significant correlation with the QFT-GIT_1_ (r = 0.150, p = 0.206). For binary results (positive or negative), the concordance rate of the QFT-GIT_1_ and the QFT-Plus_1_ was 83.6%, whereas the discordance rate was 16.4% ([Table S2 in supplement file]). The discordant QFT-Plus (+)/QFT-GIT(−) cases were younger (45.9 vs. 58.8 years, p = 0.017) but had similar T1/T2 responses (p = 0.250 and 0.221, respectively) to those of the concordant QFT-Plus (+)/QFT-GIT(+) group (Table S3 in supplement file). In the discordant QFT-Plus (−)/QFT-GIT(+) subgroup, the QFT response was 0.58 IU/ml, lower than that of the positive concordant subgroup (p = 0.027).

### Concordance of first and second follow-ups of QFT-GIT and QFT-Plus

**Figure 2:**
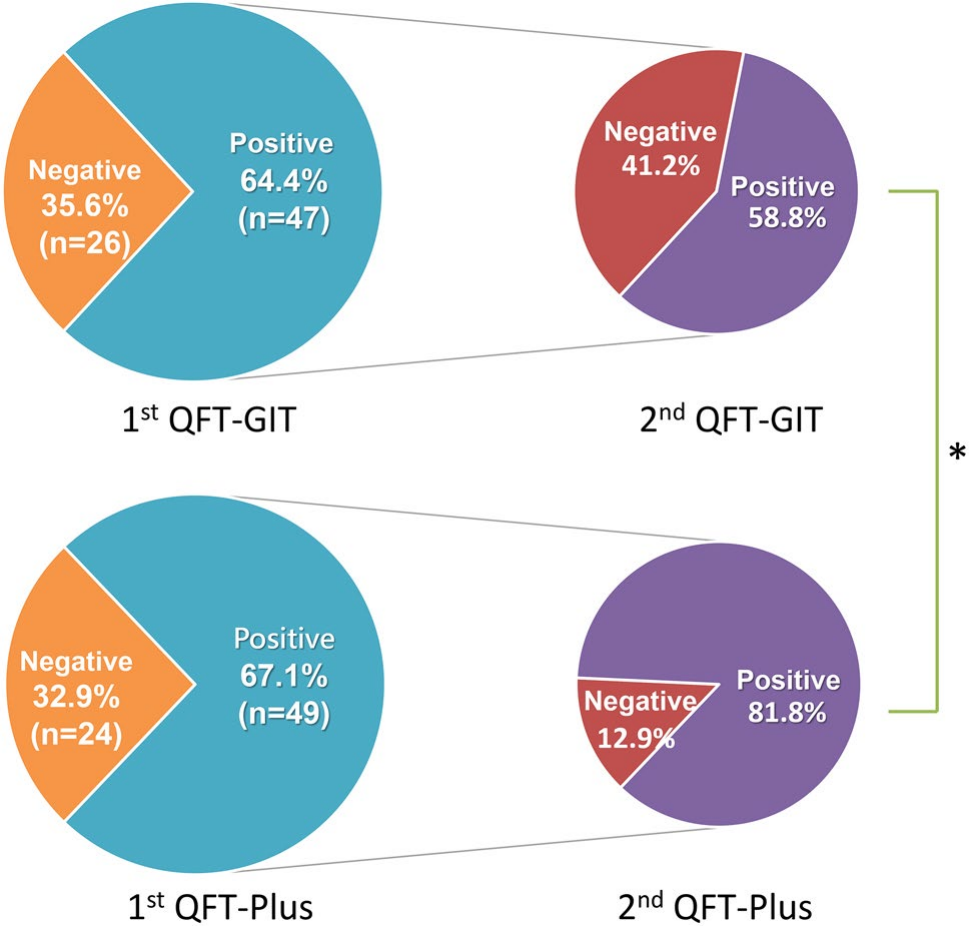
The results of the second follow-up QuantiFERON-TB tests for the subjects with positive results on the first paired QuantiFERON-TB Gold In-tube (QFT-GIT) and QuantiFERON-TB Gold plus (QFT-Plus), respectively. *p value was 0.040 for positive 2nd QFT-GIT versus QFT-Plus.

A total of 53 subjects (34 QFT-GIT_1_ positive and 33 QFT-Plus_1_ positive) completed the second follow-up round of paired QFT-GIT and QFT-Plus tests (Table 3 and Fig. 2). Persistent positivity was found in 20 subjects by the QFT-GIT (58.8%) and in 27 by the QFT-Plus (81.8%, p = 0.040). The Cohen’s kappa values of the QFT-GIT and QFT-Plus were 0.34 and 0.61, respectively. Negative concordances of the QFT-GIT and QFT-Plus were similar (78.9% vs. 80%). Even after the exclusion of 1 patient who had old TB and completed the two rounds of tests, the QFT-Plus test had better positive consistency than the QFT-GIT test (81.3% vs. 58.8%, p = 0.047).

**Table 3:** The intra-test discordance and concordance of serial QuantiFERON-TB Gold Plus (QFT-Plus) and QuantiFERON-TB Gold In-tube (QFT-GIT) tests, respectively.

(A)		
	1st QFT-Plus(−) (n = 20)	1st QFT-Plus(+) (n = 33)
2nd QFT-Plus(−)	16 (80%)	6 (12.9%)
2nd QFT-Plus(+)	4 (20%)	27 (81.8%)*
Persistent positive rate: 81.8%		
Cohen’s kappa value: 0.61		

(B)		
	1st QFT-GIT(−) (n = 19)	1st QFT-GIT(+) (n = 34)
2nd QFT-GIT(−)	15 (78.9%)	14 (41.2%)
2nd QFT-GIT(+)	4 (21.1%)	20 (58.8%)*
Persistent positive rate: 58.8%		
Cohen’s kappa value: 0.34		

*p = 0.040 by comparing persistent positivity between the 1st and 2nd QFT-GIT(+) versus the 1st and 2nd QFT-Plus(+) using Pearson chi-squared test.

### Demographic differences between persistent positivity and negative reversion patients

For the patients with persistent positive results on two subsequent QFT_2_ tests, no significant differences from their corresponding negative reversion groups existed in age, dialysis type, diabetes mellitus, BCG vaccination scar or LTBI treatment rate (Table 4). However, more men had persistent positive QFT-Plus results than negative reversion (77.8% vs. 33.3%, p = 0.053). The baseline test of the QFT-GIT_0_ (2.47 ± 0.55 vs. 1.20 ± 0.81, p < 0.001) (Table 4) and that of the QFT-GIT_1_ (3.68 ± 2.47 vs. 0.98 ± 0.81, p < 0.001) were higher in patients with persistent positivity than in those with negative reversion of QFT-GIT_2_. The T1 and T2 responses of the QFT-Plus_1_ were also higher in those with persistent positivity than in the negative reversion group. But the T2–T1 levels of subsequent QFT-Plus_1_ tests were not significantly different between the persistent positivity and negative reversion groups.

**Table 4:** Demographic data between persistent positive result or negative reversion groups of QFT-GIT and QFT-Plus.

	1st QFT-GIT(+) 2nd QFT-GIT(+) (n = 20)	1st QFT-GIT(+) 2nd QFT-GIT(−) (n = 14)	p	1st QFT-Plus(+) 2nd QFT-Plus(+) (n = 27)	1st QFT-Plus(+) 2nd QFT-Plus(−) (n = 6)	p
Age	57.7 ± 12.2	55.8 ± 10.7	0.631	55.9 ± 12.2	61.3 ± 10.4	
Sex, male n (%)	15 (75)	10 (71)	1	21 (78)	2 (33)	0.053
Dialysis (HD) n (%)	13 (65)	9 (64)	1	19 (70)	5 (83)	1
DM, n (%)	6 (30)	3 (21)	0.704	8 (30)	2 (33)	1
Previous TB n (%)	0	0	NA	1 (4)	0	NA
BCG scar n (%)	16 (80)	13 (93)	0.379	21 (78)	6 (100)	0.563
LTBI treatment n (%)	17 (85)	10 (71)	0.410	19 (70)	6 (100)	0.160
QFT-GIT_0_*	2.47 ± 0.55	1.20 ± 0.81	< 0.001	2.25 ± 0.43	2.86 ± 3.56	0.924
QFT-GIT_1_*	2.80 ± 2.37	0.98 ± 0.81	< 0.001	2.39 ± 2.22	0.63 ± 0.21	< 0.001
**QFT-Plus**_**1**_						
T1 of QFT-Plus_1_*	2.32 ± 2.15	0.80 ± 0.98	0.010	2.13 ± 1.96	0.55 ± 0.30	< 0.001
T2 of QFT-Plus_1_*	2.43 ± 2.18	0.95 ± 1.26	0.018	2.27 ± 2.01	0.62 ± 0.28	< 0.001
T2–T1 of QFT-Plus_1_*	0.11 ± 0.56	0.15 ± 0.37	0.804	0.14 ± 0.59	0.08 ± 0.06	0.563
QFT-GIT_2_*	2.17 ± 1.66	0.18 ± 0.09	< 0.001	1.67 ± 1.63	0.05 ± 0.14	< 0.001
**QFT-Plus**_2_*						
T1 of QFT-Plus_2_*	1.63 ± 1.35	0.18 ± 0.21	< 0.001	1.31 ± 1.27	0.02 ± 0.13	< 0.001
T2 of QFT-Plus_2_*	1.39 ± 0.82	0.19 ± 0.17	< 0.001	1.10 ± 0.77	− 0.01 ± 0.23	< 0.001
T2–T1 of QFT-Plus_2_*	− 0.24 ± 0.78	0.01 ± 0.11	0.163	− 0.21 ± 0.65	− 0.02 ± 0.17	0.213

QFT-GIT response: the interferon-gamma level of the TB antigen tube minus the negative control in the QFT-GIT test. The unit is IU/ml.

T1/T2 of QFT-Plus: the interferon-gamma level of the TB1 or TB2 tube minus the negative control in the QFT-Plus test. The unit is IU/ml.

T2–T1 of QFT-Plus_1_: the level of the T2 tube minus the T1 tube in the QFT-Plus test. The unit is IU/ml.

*Subscripts: 0, the baseline test; 1, the 1st follow-up; 2, the 2nd follow-up.

BCG, Bacillus Calmette-Guérin vaccine; DM: Diabetes mellitus; F, female; HD, Hemodialysis; LTBI, latent tuberculosis infection; M, male; NA, non-applicable; QFT-GIT: QuantiFERON-TB Gold In-tube; QFT-Plus: QuantiFERON-TB Gold Plus; TB, tuberculosis.

For patients with persistent positive results of the QFT-Plus_2_, their QFT-GIT_1_, T1 and T2 of the QFT-Plus_1_ were significantly higher than those with reversion (2.39 ± 2.22 vs. 0.63 ± 0.21, p < 0.001; 2.13 ± 1.96 vs. 0.55 ± 0.30, p < 0.001; and 2.27 ± 2.01 vs. 0.62 ± 0.28, p < 0.001, respectively). But the values of the baseline QFT-GIT_0_ tests were not significantly different between the persistent positivity and negative reversion groups (2.25 ± 0.43 vs. 2.86 ± 3.56, p = 0.924). As for the QFT-GIT, there were no significant differences between T2–T1 of the QFT-Plus_1_ and the QFT-Plus_2_ persistent positivity and negative reversion results.

## Discussions

To examine the positive consistency of the new QFT-Plus test, we screened 236 patients on dialysis. Among them, 73 were enrolled and 53 completed the second round of follow-up paired QFT-GIT and QFT-Plus tests in the present study. The correlation between the two tests when they were administered concurrently was excellent (Pearson’s correlation > 0.9). The negative reversion rate of the QFT-GIT was as high as 41.2% at the 6-month follow-up, higher than that of the QFT-Plus (12.9%, p = 0.040). Higher first-round IFN-γ levels of both the QFT-GIT and the QFT-Plus were significantly correlated with persistent positivity at the follow-up.

A diagnosis of LTBI is defined as a positive result from an immune assay for M.tb after active TB exclusion and based on the life-long presence of M.tb infection without adequate anti-TB treatment^21,22^. Negative reversion of the immune assay for M.tb antigens may indicate regression of the immune response, especially in immunocompromised patients with underlying comorbidities^13,15^. In our previous study, QFT-GIT negative reversion was found to be as high as 45.9% in patients on dialysis^13^, which is similar to that in the present study (41.2% by QFT-GIT). This inconsistent finding is concerning for LTBI screening among high-risk populations who have no clear history of TB exposure that could serve as a timepoint for commencement of LTBI survey. Furthermore, random LTBI testing to screen the dialysis population with QFT-GIT may produce false negative results if the immune response has fallen below the cut-off value of the immune test. Hence, optimal LTBI screening tests with high consistency would provide more reliable information for LTBI management in high-risk groups.

The positive consistency of the QFT-Plus at follow-up was higher (81.8%) than that of the QFT-GIT (58.8%), which might be explained by the greater sensitivity of the QFT-Plus in detecting LTBI consistently. For example, in the first round of the QFT-GIT_1_(−) tests, the QFT-Plus_1_ detected specific cases with a high response (T1: 0.98 IU/ml [Table S3]) beyond the detection grey zone (< 0.8 IU/ml) proposed by Metcalfe et al^23^. In addition, the difference in QFT-GIT_0_ between the QFT-Plus_1_(+) and (−) results was larger than that between the QFT-GIT_0_(+) and (−) results, indicating that the QFT-Plus might have a better discriminating ability. In addition, CD8 antigens were added to the QFT-Plus to augment CD8 effector or memory responses in individuals with defective or low CD4 responses. However, the CD8 response was not significantly different between the persistent positivity and negative reversion groups. By contrast, the CD4 (TB1 tube) or CD4 plus CD8 (TB2 tube) responses were both well correlated with persistent positivity (Table 4). Therefore, the present study indicates that remote or chronic M.tb infection might be more highly correlated with the CD4 response than with the CD8 response^24^. In this cohort of patients on dialysis, the CD4 antigens of the QFT-Plus, including ESAT 6 and CFP10 peptides but no TB7.7, as compared with the QFT-GIT, might be the dominant and consistent inducers of the memory CD4 T cell response. Our study demonstrates a more reliable response of M.tb-specific CD4 T cell response over time for the QFT-Plus than for the QFT-GIT. Even though both tests measure the CD4 response, it was reported that the QFT-Plus might be more useful in detecting LTBI in elderly^18^ and immunocompromised patients^25^. However, determining the exact mechanism of why the QFT-Plus can maintain the positive consistency well will require further study and large-scale validation.

It should be noted that the response decreased from the first to the second round, regardless of which QFT assay was used, indicating a decaying immune response to M.tb antigens. This finding is consistent with previous reports that high initial values might predict positive consistency^13^, but so far, the results have been too inconclusive to titrate a cut-off value. Because follow-up is time-consuming, our previous report also showed that additional inflammatory markers at the baseline could be used to discriminate persistent positivity from negative reversion, at a possible cost of more than one test^26^. The present study indicates that an initial positive QFT-Plus result has a likelihood of being consistent at 6-month follow-up of higher than an 80%, so this test could be considered as a one-time test.

Regarding the results of the QFT-GIT_0_, QFT-GIT_1_ and QFT-Plus_1_, respectively, the positive consistency rates were both around two-thirds, with no inter-assay difference (67% vs. 64%). Because of a lack of baseline QFT-Plus data, the QFT-GIT_0_ might be specific only for QFT-GIT_1_ follow-up. This might indicate that initial screening is a critical point in the positive judgement and has the same importance as its detection consistency. Another issue is that we recruited several patients with old TB cases, who might not have been candidates for LTBI screening. We only aimed to test the positive consistency by IGRA assay. When the results were re-analyzed for the patients without a history of old TB, the positive consistency of the QFT-Plus was still higher than that of the QFT-GIT.

This study had several limitations. First, the sample size in the present study was not large. In addition, the study design was not a randomized comparison. However, the two assays were compared using the same blood samples so as to avoid inter-subject variation. Third, the subjects were recruited from a set of patients with positive IGRA tests, so the results could not be used to interpret positive conversion from negative results. Fourth, we did not enroll patients with tuberculosis or household contacts. Further application of the CD8 response in the QFT-Plus might have a potential for disease status discrimination^27^. No participants had a history of recent TB contact, which could have affected the performance of the QFT-Plus^28^, especially the CD8 response^29,30^.

In conclusion, the present study demonstrates that the QFT-Plus had a higher positive result consistency (81.8%) at a 6-month follow-up as compared with the QFT-GIT. The inter-assay correlation was remarkably high. Another finding was that the CD4 response of the QFT-Plus might be important for positive consistency of LTBI. Although larger studies are needed to further validate the findings, we conclude that the new QFT-Plus has more reliable positive results than those of the QFT-GIT and is therefore more suitable for TB screening of dialysis patients.

## Methods

### Participants

Subjects were recruited from two tertiary-care medical centers in northern Taiwan in 2016–2019 under IRB approval (Nos. 201603066DIPB and 108067-F). Written informed consent was obtained. Patients on maintenance dialysis underwent LTBI screening according to the routine health policy of one hospital and by invitation in a research survey at the other. The LTBI screening included chest plain film and the QFT-GIT. If radiography indicated possible TB, three sets of sputum cultures for M.tb were obtained. Patients on dialysis with positive QFT-GIT results but no evidence of active TB were included. Patients with diagnoses of human immunodeficiency virus infection or active TB were excluded. Identifiers of participants were removed in accordance with the Health Insurance Portability and Accountability Act (HIPAA) Privacy Rule. We confirmed that all experiments were performed in accordance with relevant guidelines and regulations.

### Study design

After a participant had been enrolled, the QFT-GIT and QFT-Plus (QIAGEN, Germany) were performed concurrently on the same blood sample according to the manufacturer’s instructions^17,31^. The interferon-gamma (IFN-γ) level of the post-reaction supernatant was then measured with an enzyme-linked immuno-sorbent assay. The IFN-γ response of the QFT kits was the IFN-γlevel in the TB antigen tube minus that in the negative-control tube in the QFT-GIT and the TB1 or TB2 tube minus the negative-control tube in the QFT-Plus. Results were interpreted as positive, negative, or indeterminate^32,33^. Around 6 months later, the QFT-GIT and QFT-Plus were followed up together on the same participants and on the same samples of whole blood. Regarding the primary end-point, we compared the proportions of persistent positive results (consistency) of the different QFT kits.

### Data collection

We recorded participant’s demographics, including age, gender, and comorbidities, as well as dialysis type (hemodialysis or peritoneal dialysis). Standardized case report forms were used and completed with default options. The Bacillus Calmette Guérin (BCG) vaccination scar was checked. The IFN-γ levels of the QFT-GIT and QFT-Plus were recorded.

### Statistical analyses

Pearson’s chi-squared test or Fisher’s exact test was used to compare categorical variables, and continuous variables were compared using the Student’s t test or the Mann–Whitney U test, where appropriate. Pearson’s correlation analysis was used to assess relationships between the first and second pairs of the QFT-GIT and QFT-Plus assays. All analyses were performed in SPSS software (version 19.0 for Windows, SPSS Inc.).

### Ethics approval and consent to participate

The Research Ethics Committee of National Taiwan University Hospital (IRB No.: No. 201603066DIPB) and the Research Ethics Committee of Far-Eastern Memorial Hospital (No. 108067-F) approved this study.

## Consent for publication

Written informed consent was obtained from the participants at the time of enrollment.

## Supplementary Information

Supplementary Information.
