# Supplementary material for: CD4 response of QuantiFERON-TB Gold Plus for positive consistency of latent tuberculosis infection in patients on dialysis
Source: Sci Rep. 2020 Dec 7;10:21367. doi: 10.1038/s41598-020-78374-3 (PMC7721715; doi:10.1038/s41598-020-78374-3)
Supplement: Supplementary file 1 — Supplementary Information. [file 41598_2020_78374_MOESM1_ESM.docx]

*Original Article*

**CD4 response of QuantiFERON-TB Gold Plus for positive consistency of latent tuberculosis infection in patients on dialysis**

Pin-Huai Wang^1,2^, Shu-Yung Lin^3,4^, Susan Shih-Jung Lee^5^, Shu-Wen Lin^6^, Chih-Yuan Lee^4,7^, Yu-Feng Wei^8^, Chin-Chung Shu^3,4^, Jann-Yuan Wang^3,4^, Chong-Jen Yu^3,4^

**Institutions:**

^1^Division of Pulmonology, Department of Internal Medicine, Far Eastern Memorial Hospital, New Taipei City, Taiwan

^2^ Department of Nursing, Oriental Institute of Technology, New Taipei City, Taiwan

^3^Department of Internal Medicine, National Taiwan University Hospital, Taipei, Taiwan

^4^College of Medicine, National Taiwan University, Taipei, Taiwan

^5^Division of Infectious Diseases, Department of Internal Medicine, Kaohsiung Veterans General Hospital, Kaohsiung, Taiwan; Faculty of Medicine, School of Medicine, National Yang-Ming University, Taipei, Taiwan.

^6^Graduate Institute of Clinical Pharmacy, National Taiwan University, Taipei, Taiwan

^7^Department of Surgery, National Taiwan University Hospital, Taipei, Taiwan

^8^Division of Chest Medicine, Department of Internal Medicine, E-Da Hospital, Kaohsiung city, Kaohsiung, Taiwan

**Corresponding authors**:

Chin-Chung Shu, MD, PhD.

Department of Internal Medicine, National Taiwan University Hospital, Taipei, Taiwan Address: No 7, Chung Shan South Road, Taipei, Taiwan; TEL: +886223123456#62905

Email: ccshu@ntu.edu.tw

**Running Title:** LTBI consistency by QuantiFERON-TB in dialysis patients

**Table S1.** Differences in baseline TB tube responses between persistent positive and negative reversion results of 1^st^ follow up of the QFT-GIT and QFT-Plus

| QFT_0-1_ | persistent positive | negative reversion | p |
| --- | --- | --- | --- |
| QFT-GIT_1_ | n=49 | n=24 |  |
| TB-Nil_0_ | 2.65 ± 2.26 | 1.60 ± 2.56 | 0.080 |
| QFT-Plus_1_ | n=47 | n=26 |  |
| TB-Nil_0_ | 2.93 ± 2.38 | 1.18 ± 2.02 | 0.002 |

Abbreviations: QFT_0-1:_ QuantiFERON-TB test, baseline result versus 1^st^ test ; QFT-GIT: QuantiFERON-TB Gold In-Tube test; QFT-Plus: QuantiFERON-TB Gold Plus; TB-Nil: the interferon-gamma level of the TB antigen tube minus that in the negative control tube; subscript 0 or 1: baseline or 1^st^ follow-up of the test

**Table S2.** **Discordance and concordance of** **first follow-up QFT-Plus and QFT-GIT tests.** In a total of 73 subjects that received the 1^st^ pair of QFT-GIT and QFT-Plus tests, the concordance rate of the QFT-GIT and the QFT-Plus was 83.6% and the discordance rate was 16.4%. Pearson correlation of the two tests was 0.636 (p < 0.001) and Cohen’s Kappa coefficient was 0.635.

|  | QFT-G-GIT_1_(+) | QFT-G-GIT_1_(-) |
| --- | --- | --- |
| QFT-Plus_1_(+) | 42 | 5 |
| QFT-Plus_1_(-) | 7 | 19 |

Abbreviations: QFT-G-GIT: QuantiFERON-TB Gold In-Tube; QFT-Plus: QuantiFERON-TB Gold Plus; Subscript 1: the first follow-up tests

**Table S3.** Demographic data and interferon response levels to TB antigen among concordance and discordance of first follow-up QFT-GIT and QFT-Plus tests

|  | QFT-G-GIT_1_(+)  QFT-Plus_1_(+) | QFT-G-GIT_1_(+)  QFT-Plus_1_(-) | P1 value | QFT-G-GIT_1_(-)  QFT-Plus_1_(+) | P2 value | QFT-G-GIT_1_ (-)  QFT-Plus_1_ (-) |
| --- | --- | --- | --- | --- | --- | --- |
| **Number** | 42 | 7 |  | 5 |  | 19 |
| **Age** | 58.8 ± 11.0 | 51.5 ± 10.6 | 0.110 | 45.9 ± 10.7 | 0.017 | 56.9 ± 10.6 |
| **Sex, male (%)** | 30 (71.4) | 5 (71.4) | 1 | 3 (60) | 0.627 | 14 (73.7) |
| **QFT-GIT_0,_ IU/ml** | 2.84 ± 2.25 | 1.52 ± 2.16 | 0.155 | 3.67 ± 3.56 | 0.467 | 1.06 ± 2.01 |
| **QFT-GIT_1,_ IU/ml** | 2.43 ± 2.15 | 0.58 ± 0.19 | 0.027 | 0.23 ± 0.16 | 0.028 | 0.08 ± 0.20 |
| **QFT-Plus_1_** |  |  |  |  |  |  |
| **T1 of QFT-Plus_1,_ IU/ml** | 2.06 ± 2.03 | 0.23 ± 0.94 | 0.022 | 0.98 ± 0.93 | 0.250 | 0.08 ± 0.20 |
| **T2 of QFT-Plus_1,_ IU/ml** | 2.18 ± 2.18 | 0.25 ± 0.061 | 0.024 | 0.96 ± 0.62 | 0.221 | 0.07 ± 0.20 |
| **T2-T1 of QFT-Plus_1,_ IU/ml** | 0.12 ± 0.65 | 0.015 ± 0.083 | 0.682 | -0.02 ± 0.57 | 0.652 | -0.015 ± 0.053 |

Abbreviations: QFT-G-GIT: QuantiFERON-TB Gold In-Tube; QFT-Plus: QuantiFERON-TB Gold Plus; Subscript 0 or 1: the baseline or the first follow-up test, respectively; T1: TB1 antigen tube; T2: TB2 antigen tube

P1 value compares patients with QFT-G-GIT_1_(+)/QFT-Plus_1_(+) and those with QFT-G-GIT_1_(+)/QFT-Plus_1_ (-), and P2 value compares patients with QFT-G-GIT_1_(+)/QFT-Plus_1_(+) and those with QFT-G-GIT_1_(-)/QFT-Plus_1_(+).
